# Chronic Psychological Stress, but Not Chronic Pain Stress, Influences Sexual Motivation and Induces Testicular Autophagy in Male Rats

**DOI:** 10.3389/fpsyg.2020.00826

**Published:** 2020-04-30

**Authors:** Yunyun Shen, Danni He, Luhong He, Yu Bai, Bo Wang, Yan Xue, Gonglin Hou

**Affiliations:** Institute of Cognitive Neuroscience and Department of Psychology, College of Science, Zhejiang Sci-Tech University, Hangzhou, China

**Keywords:** chronic stress, chronic pain, testis, autophagy, sexual motivation

## Abstract

Spermiogenesis is an important physiological process of mammalian fertilization. The germ cells are susceptible to the harmful effects of either psychological or physiological stress, which could induce male infertility. Our previous studies have found that chronic psychological stress could decrease sexual motivation. However, molecular mechanisms underlying male reproductive toxicity induced by chronic stress remain elusive. Recently, autophagy is proven to be involved in regulating the survival of germ cells, which is related to apoptosis. Herein, we established a chronic psychological stress model and a chronic pain model (physiological stressor) to explore the roles of autophagy in germ cells. Thirty-two male Sprague-Dawley rats were randomly divided into four groups, including the control group, the chronic psychological stress group, the SNI-sham group, and the chronic pain stress group. After exposure to stress for 35 days, open field test and the unconditioned sexual motivation test were performed. Following the behavioral experiment, autophagy in the rat testis was detected by Western blot and immunohistochemistry. We found both chronic psychological stress and chronic pain stress reduced total travel distance, the frequency of central crossing and increased the sensitivity to mechanical pain. While chronic psychological stress, but not the chronic pain stress declined sexual motivation. Chronic psychological stress prompt the expression of LC3-II with the decreased expression of p62, indicating that chronic psychological stress induced autophagy in rat testis. However, there was no significant difference between the expression of LC3-II and p62 in male rats under chronic pain stress. Therefore, chronic psychological stress and chronic pain stress have common behavior changes, but due to its unpredictability, chronic psychological stress leads to a decline in sexual motivation in male rats and induced the autophagy in testicular tissues.

## Introduction

A declining trend in sperm concentration over the past 35 years with perceptions of the reasons of such deterioration in male reproductive health (Sengupta et al., 2017). Chronic stress triggers a series of cognitive dysfunction, metabolic syndrome, cardiovascular diseases, immune system dysfunction (Mulroney and Tache, 2010; Turdi et al., 2012; Lupien et al., 2018), and also affect the reproductive system which could further cause male infertility (Ebbesen et al., 2009; Garcia-Diaz et al., 2015). Recent studies showed that chronic stress causes germ cell loss and apoptosis in testes, possibly as a result of testosterone decrease, affecting fertility (Almeida et al., 1998; Kanter et al., 2013; Retana-Marquez et al., 2014). However, apoptosis is closely related with autophagy which plays a role in various physiological functions such as intracellular pathogens clearance so as to protect the organism from various diseases. A study of autophagy in the reproductive system of rats found that defective autophagy and excessive apoptosis in the aggravation of testicular damage (Zhang et al., 2016). Therefore, we speculated that impaired autophagy after chronic stress exposure could have a potential correlation with reproductive health.

Chronic stress could induce autophagy in hippocampus, bone marrow, nerves, and synaptic regeneration, etc. However, few studies have focused on exploring changes in the autophagy activity of the reproductive system and the mechanism merit further investigation. In our daily life, the most common stressors could be simply divided into psychological stress and physical stress. Clinically, these two types of stress are prone to comorbidity, such as anxiety, depression and chronic pain. Therefore, comparing the effects of different sources of stress on reproductive system would supply a profound understanding of the pathogenic mechanisms of stress in male infertility.

Our previous study found that chronic unpredictable mild stress (CUMS) could trigger organic damage to testicular cells in male rats in correlation with alterations in sexual preference behaviors (Hou et al., 2014). In the present study, we aim to further investigate the effect on testicular germ cell autophagy and apoptosis by chronic psychological stress and chronic pain stress model.

## Materials and Methods

### Animals

A total of 34 male and 4 female Sprague-Dawley rats (Shanghai Laboratory Animal Center at the Chinese Academy of Sciences, Shanghai, China) weighing 230 ± 10 g were housed under temperature- and humidity-controlled (22 ± 2°C, 50–60% humidity) rooms and supplied with food and water ad libitum. Thirty-two male rats were randomly divided into a control group, a chronic psychological stress group, a SNI-sham group, and a chronic pain group. The chronic psychological stress group and the chronic pain group were established by the chronic unpredictablemild stress model and the spared nerve injury (SNI) model, respectively. Rats were placed in the rooms 7 days prior to testing in order to ensure adaptation to the environment (12-h light, 12-h dark cycle, lights on 8 a.m.) and habituation to handling. Two male and four female Sprague-Dawley rats were prepared for the unconditioned sexual motivation test. All animal protocols were approved by the Animal Care and Use Committee for the Department of Psychology at Zhejiang Sci-Tech University in accordance with NIH guidelines for the care and use of laboratory animals.

### Chronic Unpredictable Mild Stress Model

The experimental procedure was adapted from that described by Hou et al. (2015). Briefly, male rats were randomly separated into the control group (*n* = 8) which were remained undisturbed in their cages, whereas the rats in the stress group (*n* = 8) were housed separately and were exposed to CUMS for 35 days. Seven stressors (a tail clamp stimulus, wet bedding, electric foot shock, cold water immersion, food deprivation, water deprivation, and reversed light/dark cycle) were applied in a random order for 35 consecutive days during the light phase. The experiment was conducted in accordance with the National Animal Welfare Standards and codes of ethics.

### Spared Nerve Injury Model

This study used the sciatic nerve branch selective impairment (spared nerve injury, SNI) model for neuropathic pain study (Richner et al., 2011). Briefly, the main steps are brief described as follows: (1) anesthetize rats and cut open the outer epidermis of the thigh, and exposing the sciatic nerve and its branches by blunt separation including fiphointestinal nerve, common peroneal nerve and tibial nerve. (2) Tightly knot the common peroneal and tibial nerve with 5.0 wire, the SNI-sham group involved only the exposure of the sciatic nerve and its branches without causing any damage. 3) The muscle layer is then closed, then the wound is stitched and disinfected.

### Estrous Cycle Determination

The estrous cycle phases of female rats were determined as previously described (Hou et al., 2014). Briefly, vaginal secretion was collected with a cotton swab and placed on glass slides. The vaginal secretions of the four female rats were collected and observed twice per day under microscopy at 8 a.m. and 10 p.m. Determination of estrous cycle phase was based on the two complete estrous cycles observed before the test day by the characteristics of the vaginal secretions of female rats.

### The Unconditioned Sexual Motivation Test

The apparatus for the test of the unconditioned sexual motivation was described as previously (Agmo, 2003; Hou et al., 2014). The time spent in incentive zones, the number of visits to the zones, and the total distance traveled were monitored and recorded by Noldus EthoVision XT (Noldus, Netherlands). First, the subjects were familiarized with the test arena for 3 days, 10 min/day, without incentive rats in cages. Before each test, the arena and the cages were carefully cleaned with 75% alcohol. Then, the incentives (one female and one male rat) were placed in their cages. About 5 min later, the rat was introduced into the middle of the arena and observed for 10 min. The unconditioned sexual motivation was quantified by the preference score [time spent in the female incentive zone/(time spent in the female incentive zone + time spent in the male incentive zone)].

### Open Field Test

The uncapped open box (80 cm × 80 cm × 40 cm) is used as a field test device, and the central grid is limited to a range of 40 cm × 40 cm in the central position. Each rat was then placed in the center of the uncapped open box, allowing freely movement for 5 min. The behavior was recorded by a video system. At the end of each rat experiment, clean the waste from the field with a clean paper towel and spray 75% of the alcohol to remove the residual odor. The frequency of rearing and central crossing, the latency time in the central zone and total travel distance were analyzed by Noldus software EthoVision XT.

### Von Frey Test

According to previous study (Richner et al., 2011; Pitzer et al., 2016), each rat was placed separately in a cage to accommodate for 15min. Then, Von Frey fibers with varying diameters were used to test the rat’s sensitivity to a mechanical stimulus, and each bending force was measured five times. The number of withdrawals was recorded. When the number of withdrawals reached 40% (2 out of 5), paw withdrawal mechanical threshold (PWMT) was recorded. Pain sensitivity tests of both feet were performed on all rats per week.

### Histology

Briefly, after blocking with 0.5% BSA (Bovine serum albumin) in PBST (Phosphate Buffered Saline with Tween 20), paraffin-embedded testis slices were incubated with LC3 antibodies at 4°C overnight (Anti-LC3, MBL Inc, #PM036). Biotin-conjugated secondary antibodies (CST, #8114) and ABC-DAB staining kit (Vecta ABC and DAB standard kit, #PK-400 and SK-4105) were used for antibody localization and the nuclei were counter-stained with hematoxylin. For TUNEL staining, tissues were dewaxed and detected with Fluorometric TUNEL System (Promega) according to the working manual.

### Western Blot

Proteins were extracted from rat testes in RIPA buffer and separated by SDS-PAGE. The resolved proteins were transferred to PVDF membranes (Millipore). Non-specific reactivity was blocked in 5% bovine serum albumin for 2 h at room temperature. Diluted primary antibody (LC3B, SIGMA-ALDRICH, #L7543, diluted 1:1000; p62, CST, #5114, diluted 1:1000) was then incubated, followed by the appropriate secondary antibody. Protein detection was achieved with the ECL (Thermo Fisher Scientific). Relative protein level was calculated as a percentage of reference protein β-actin (CST, #4970, diluted 1:1000).

### Statistics

All statistics were performed with the software program SPSS 17.0 (IBM Inc., Armonk, NY, United States). Data were presented as mean ± SEM. Comparison between groups was done for statistical significance by a *t*-test. Effects were considered significant for *p* < 0.05.

## Results

### The Effect of Chronic Stress on Body Weight Gain and Anxiety-Like Behavior

**FIGURE 1:**
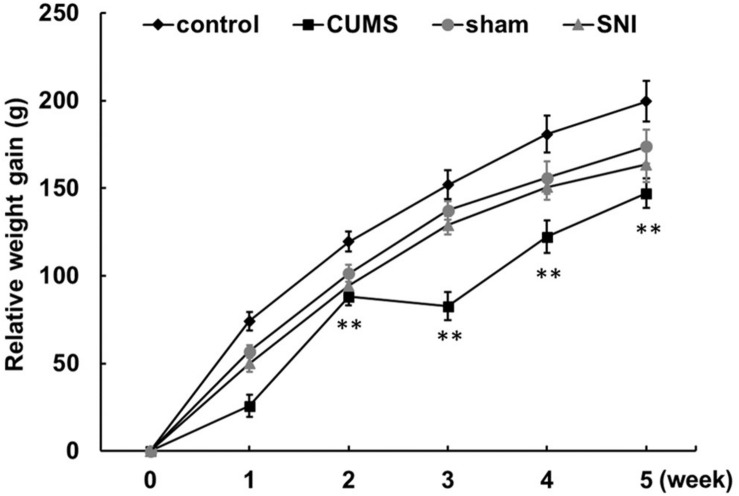
Chronic psychological stress model significantly reduces weight gain in rats. Relative weight gains were body weight per week relative to body weight at week 0. Data are shown as mean ± standard error (*M* ± SEM). *N* = 8. **p < 0.01 vs. control group.

From day 1 to day 35, the control group, the chronic psychological stress group, the SNI-sham group, and the chronic pain group were measured for body weight weekly (Figure 1 and Supplementary Figure S1). The increase in body weight compared to day 1 was calculated. The data showed that the weight gain of the psychological stress group was significantly lower than that of the control group from the first week to the fifth week, but there was no significant difference in the weekly weight gain between the SNI-sham group and the chronic pain group. It is indicated that the chronic psychological stress model significantly reduces the weight gain of rats. In addition to the weight loss caused by surgical operation, SNI did not have a significant effect on weight gain.

**FIGURE 2:**
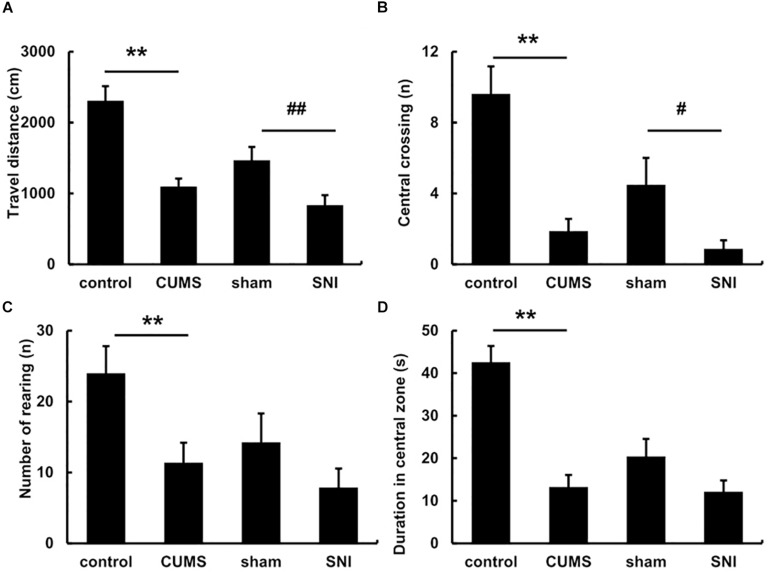
The open field behavior change in the total travel distance **(A)**, the frequency of central crossing **(B)**, number of rearing **(C)** and the duration of the central zone **(D)** after exposure to chronic stress for 5 weeks. Data are shown as mean ± standard error (*M* ± SEM). *N* = 8. **p < 0.01 vs. control group. #*p* < 0.05 vs. SNI-sham group.

The pre-test of open field experiment showed that there was no statistical difference in the total travel distance, the frequency of central crossing, number of rearing and the duration of the central zone in the four groups of rats (Supplementary Figure S2). However, after exposure to the chronic stress for 5 weeks, the post-test of open field (Figure 2) exhibited the total travel distance and number of rearing in the chronic psychological stress group was significantly reduced than the control group (*p* < 0.01), and latency time in the central zone was also significantly decreased (*p* < 0.01). Compared with the SNI-sham group, there was significant decline in the total travel distance (*p* < 0.01) and the frequency of central crossing (*p* < 0.05) in the chronic pain group, but not the number of rearing or the duration of the central zone.

### The Effect of Chronic Stress on Pain Sensitivity

**FIGURE 3:**
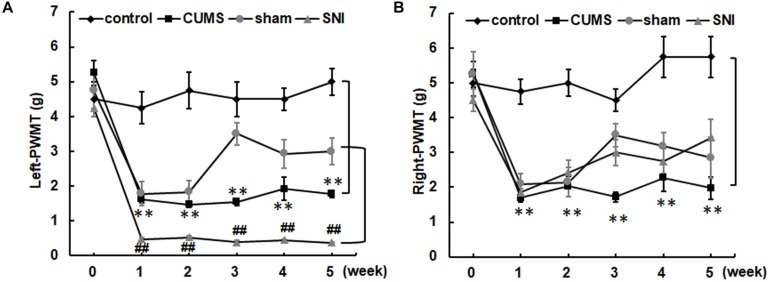
The left **(A)** and right **(B)** paw withdrawal mechanical thresholds of the four groups. Data are shown as mean ± standard error (*M* ± SEM). *N* = 8. **p < 0.01 vs. control group. ##*p* < 0.01 vs. SNI-sham group.

Before chronic stress, there was no difference in pain threshold among the four groups of rats (Figure 3). After 35 days of chronic stress, the left and right PWMTs of the chronic psychological stress group were significantly lower than those of the control group (*p* < 0.01). However, chronic psychological stress and chronic pain stress caused the threshold of mechanical pain in the left paw of rats to be significantly lower than that in the control group and SNI-sham group (*p* < 0.01). The threshold of mechanical pain in the right paw of the chronic pain group was not significantly different from that of the SNI-sham group, while the left paw (injury paw) of the chronic pain group was significantly lower than that of the SNI-sham group (*p* < 0.01), indicating that the chronic pain model was successfully established.

### The Effect of Chronic Stress on Sexual Motivation

The vaginal smear of the diestrus is mainly composed of leukocytes, while the estrus smear is primarily consisted of cornified cells (data not shown). The time between diestrus and estrus can comply with the requirements for experiments.

**FIGURE 4:**
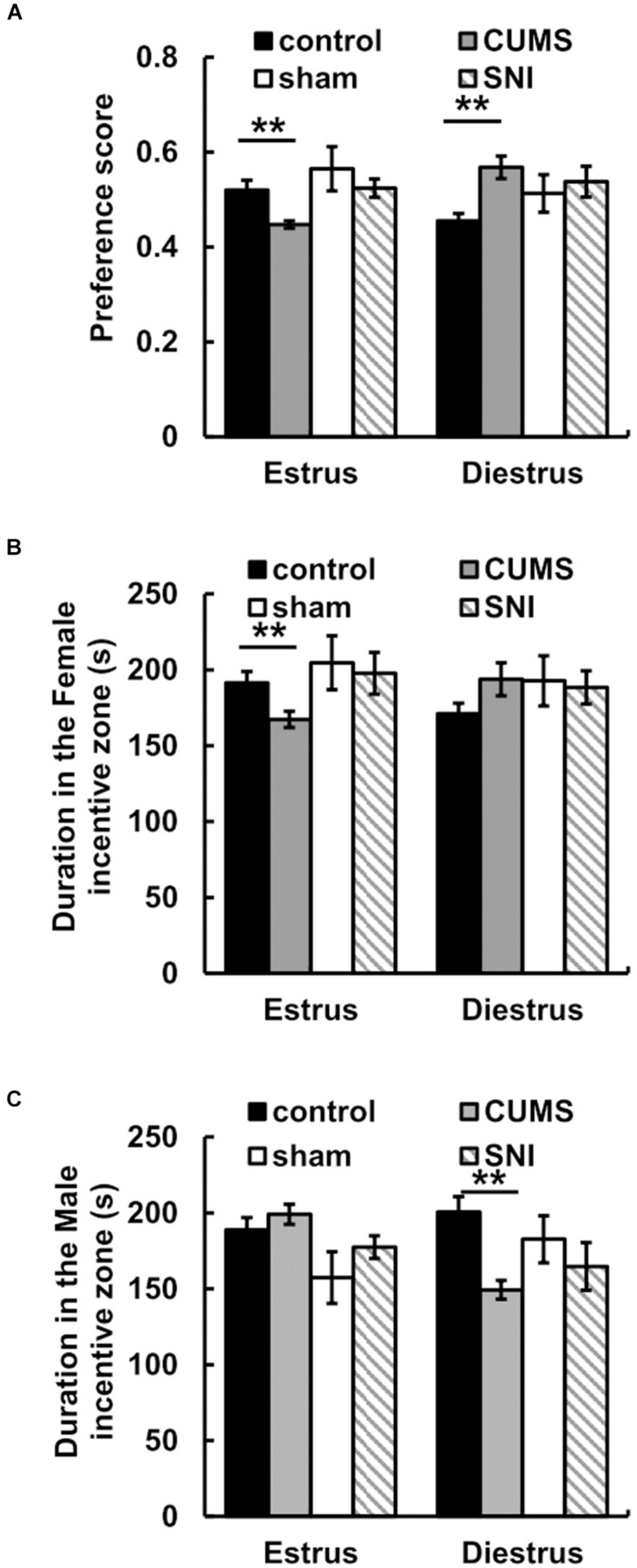
The effect of chronic stress on preference scores of estrus and diestrus rats **(A)** and sexual motivation **(B,C)**. Data are shown as mean ± standard error (*M* ± SEM). *N* ≥ 7. **p < 0.01 vs. control group.

For the preference scores in Figure 4, when female rats came into estrus, the preference score of the chronic psychological stress group was significantly reduced compared with that of the control group (*p* < 0.01). In the psychological stress group, there was significant decrease between the preference scores of male rats for estrous and diestrous female rats (*p* < 0.01), while in the control group the preference score of male rats for estrous female rats was higher than that for diestrous female rats (*p* < 0.05). When female rats came into diestrus, there was conversely significant increase in preference score between the psychological stress and control groups. In addition, the chronic pain stress did not display any obviously influence on the alteration of sexual motivation.

For the time spent in incentive zones, the results showed that there was no significant difference between the time that male rats in the stress group spent in the male zone when female rats were in estrus and the time that male rats in the stress group spent in the female zone when female rats were in diestrus (*p* > 0.05). When female rats were in estrus, less time was spent in the female incentive zone in the chronic psychological stress group compare to the control group (*p* < 0.01) (Figure 4). Meanwhile, we found that there was no difference between the time spent near the female zone in the chronic pain group and that in the SNI-sham group when female rats were in estrus or diestrus (*p* > 0.05).

### Chronic Stress Induced Autophagy in Testicular Tissues

**FIGURE 5:**
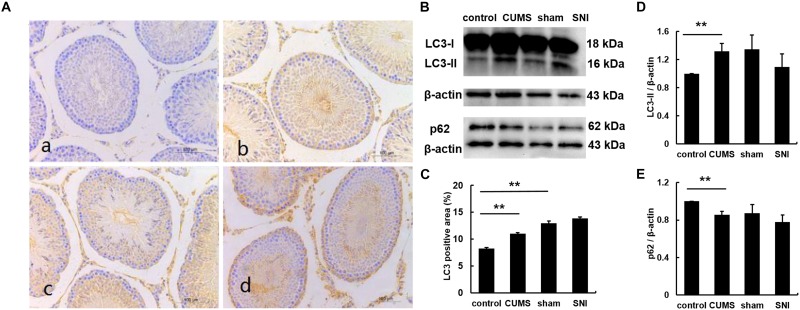
The expression of LC3B protein in the testis tissues of four groups (a, control group; b, CUMS group; c, SNI-sham group; d, SNI group) by IHC staining **(A)**, and the LC3 positive area was quantified **(C)**. The expression of LC3 and p62 in the testis tissues of four groups **(B,D,E)** by western blot. Data are shown as mean ± standard error (*M* ± SEM). *N* ≥ 6. **p < 0.01 vs. control group.

The expression of LC3 was observed by immunohistochemistry (Figure 5). The percentages of LC3 positive cells in the testes of the four groups were calculated by Image J software. The results showed that the percentage of positive cells in the chronic psychological stress group was significantly higher than that in the control group, indicating that the autophagy of the chronic psychological stress group increased, but there was no significant difference between the SNI-sham group and the chronic pain group (*p* > 0.05). Western blotting also indicated that the expression of LC3-II in the chronic psychological stress group was significantly higher than that in the control group (*p* < 0.05). Similarly, there was no significant difference in LC3-II protein expression levels between the SNI-sham group and the chronic pain group (Figure 5D). The expression of p62 in the testis of the chronic psychological stress group was significantly lower than that of the control group (*p* < 0.05) but not between the SNI-sham group and the chronic pain group (Figure 5E).

### Chronic Stress Induced the Testicular Cell Apoptosis

**FIGURE 6:**
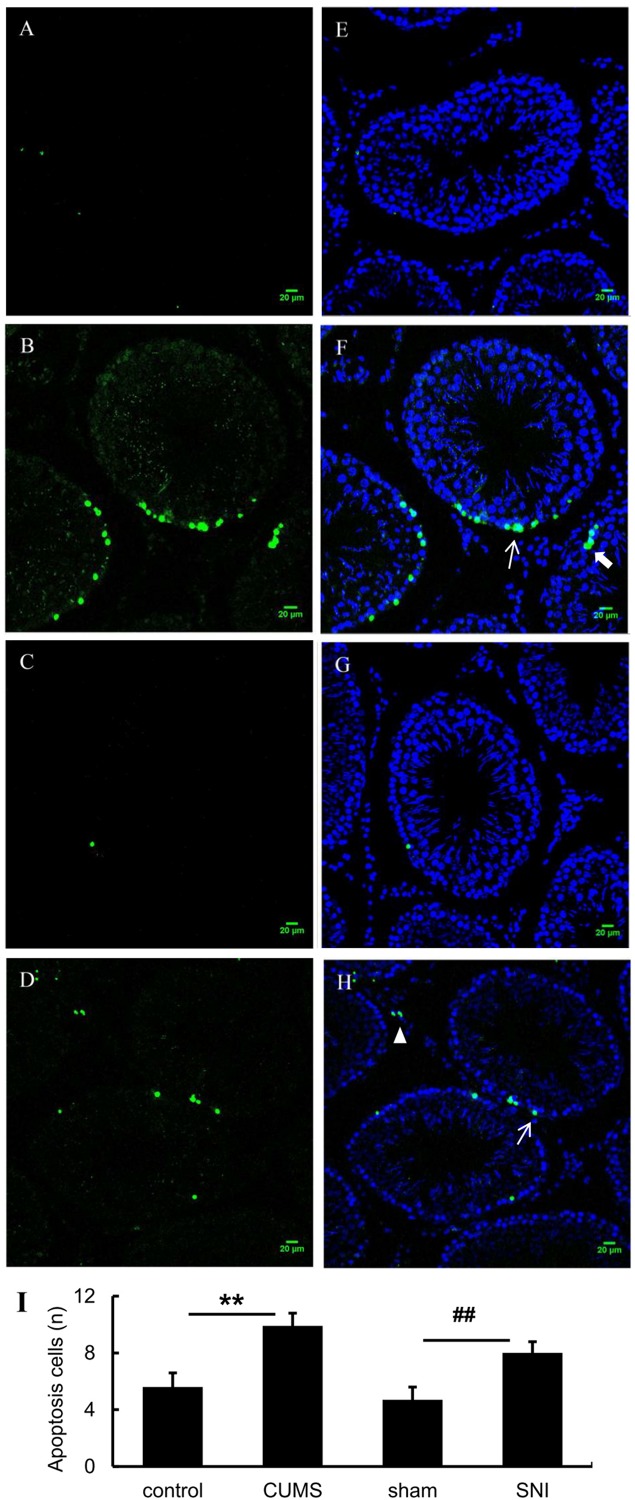
TUNEL (green) positive apoptotic cells in rat testis induced by chronic stress (**A**, control group; **B**, CUMS group; **C**, SNI-sham; **D** group, SNI group). The nuclei were counter-stained with DAPI (**E**, control group; **F**, CUMS group; **G**, SNI-sham group; **H**, SNI group). Quantitative analysis of TUNEL staining **(I)** are shown as mean ± standard error (*M* ± SEM). *N* ≥ 6. **p < 0.01 vs. control group. ##*p* < 0.01 vs. SNI-sham group.

As shown in Figures 6A–H, tunel foci of both chronic stress model groups were more obviously than those of the control group and the SNI-sham group. Apoptosis occurred in three types of cells in the testis including Leydig cells, spermatogenic cells and supporting cells, which were mainly observed in the both chronic stress groups. Descriptive statistics were performed on the number of testicular apoptotic cells in each field of the four groups of rats (Figure 6I). It was indicated that the number of testicular cell apoptosis in the chronic psychological stress group was significantly higher than that in the control group (*p* < 0.01). In addition, the number of apoptotic cells in the chronic pain group was also significantly higher than that in the SNI-sham group (*p* < 0.01).

## Discussion

Compare chronic psychological stress with chronic pain stress, two stress models were partly similar in behaviors, such as induced the sensitivity of mechanical pain threshold and anxiety-like behavior in rats. However, chronic psychological stress but not chronic pain stress attenuated male preference for the estrous female rats and generated autophagy and apoptosis in seminiferous tubules. Here, the results suggested that decreased sexual motivation under the psychological stress could not only be characterized as a kind of behavior disorder but also essentially impair male reproductive system.

Sexual motivation is one of the incentives, so we used an experimental device to detect this motivation and evaluated sexual motivation level with the preference score in rats (Agmo, 2003). In this study, the female preference scores of the rats in the chronic psychological stress group were significantly lower than those in the control group, indicating that chronic psychological stress attenuated the sexual motivation of the rats. On the other hand, the SNI-sham group and the chronic physiological stress group did not show significant differences in the female preference score and the latency time at the stimulation area, indicating that chronic pain did not affect male preference for the estrous female rats. Chronic unpredictable mild stress model due to its unpredictability mimicked different daily life stressors has a more obvious effect on behavior disorder than constant physical stress, so the outcome of the chronic psychological stress group showed that chronic psychological stress could lead to more serious stress related diseases including infertility (Hou et al., 2014; Demirci and Sahin, 2019).

Male reproductive function could be disrupted by various chronic stressors. It is reported that immobilization stress and forced swimming stress augment testicular toxicity and reduce the fertilization capacity in rats (Saki et al., 2009; Priya and Reddy, 2012). In the process of stress, autophagy is a very important metabolic pathway for the survival of the body. By studying the effects of chronic psychological stress and chronic pain stress on hippocampal autophagy, we found that autophagy was increased under chronic psychological stress while the autophagy in chronic pain group was almost constant (data not shown). Therefore, it could be speculated that different stressor participates in different ways.

An increase of LC3-II and accumulation of autophagic vesicles were found in spermatogonial cells disrupted by glutathione metabolism (Mancilla et al., 2015). Additionally, LC3-II expression was increased in 6 h, 12 h and 2 days after heat stress treatment in mouse testicular (Zhang et al., 2012). When male Sprague-Dawley rats were exposed to different doses of formaldehyde for four weeks, autophagy in testicular tissue in the high-dose treatment group was significantly increased (Han et al., 2015). In addition, intraperitoneal injections of different doses of cadmium chloride to rats for five weeks also induced an increase in LC3-II expression (Wang et al., 2017). The expression of LC3-II in the above studies is consistent with our results under the chronic psychological model, indicating that testicular LC3-II could be characterized as a common indicator of chronic stress including chemical, physical, and psychological stress.

However, the increase only in LC3-II does not indicate the activation of autophagy, because the increase in autophagosome LC3-II may be due to the fact that autophagy activation or inhibition of autophagy downstream degradation which would induce the accumulation of a large number of autophagosomes. Therefore, in the study of autophagy, the detection of autophagy substrate protein, p62, is often used as one of the hallmark autophagy degradants in autophagy (Sahani et al., 2014). p62 migrates not only to the phagocytic membrane, but also to the site of autophagosome formation. During autophagy, p62 can be selectively degraded by binding to LC3-II, so the accumulation of p62 often indicates that autophagy is inhibited. This is one of the reasons that p62 is used to detect autophagy flux (Pankiv et al., 2007). Here we found that the p62 protein in the testis of the chronic psychological stress group was significantly lower than that of the control group, but there was no significant difference in the p62 protein between the chronic pain group and the SNI-sham group. Combined with the results of the LC3 expression, it was indicated that chronic psychological stress triggered testicular autophagy in rats.

Under chronic pain stress, there was no significant difference between the rat testis LC3-II and p62 and the SNI-sham group, indicating that chronic pain did not affect the autophagy of rat testis. It was further found by immunohistochemistry that the expression of LC3B in the psychological stress group was significantly higher than that in the control group after DAB staining. There was no significant difference between the SNI-sham group and the chronic pain group, which was consistent with the results of Western blot. These data demonstrated that chronic psychological stress but not chronic physiology stress lead to a rise of autophagy in testicular tissues of male rats.

As known, some environmental toxicants, including heavy metals, pyrethroid pesticides, other endocrine disruptors, lifestyles, etc., can cause male reproductive toxicity (Sharpe, 2000; Wang et al., 2016), in which testicular histopathology is a necessary condition for assessing male reproductive toxicity. Previous studies have showed that chronic stress could lead to qualitative and functional damage to the testis, such as inhibition of spermatogenesis, specific stage of germ cell apoptosis, and testicular atrophy (Yin et al., 1997; Hjollund et al., 2004). In addition, apoptosis of testicular germ cell are different under chronic stresses. For example, primary spermatocytes and sperm cells are susceptible to heat exposure (Yin et al., 1997), and immobilization stress can enhance apoptosis of testicular spermatogenic cells (Wyllie et al., 1980). In the present study, according to the TUNEL test results, the number of apoptotic cells in the chronic psychological stress group was significantly higher than that in the control group, and the number of apoptosis in the chronic pain group was also significantly higher than that in the SNI-sham group, indicating that organic damage occurred in the reproductive system of two models. Compared with chronic psychological stress, chronic pain stress did not affect male sexual motivation and autophagy, but could affect germ cell apoptosis, indicating that the mechanisms of these two stress models acting on the reproductive system might be different.

## Data Availability Statement

All datasets generated for this study are included in the article/Supplementary Material.

## Ethics Statement

The animal study was reviewed and approved by the Animal Care and Use Committee for the Department of Psychology at Zhejiang Sci-Tech University.

## Author Contributions

GH and YS contributed conception and design of the study. YS, DH, LH, YB, BW, and YX performed the experiments. YS, DH, LH, and YB performed the statistical analysis. GH and YS wrote the manuscript. All authors contributed to manuscript revision, read and approved the submitted version.

## Conflict of Interest

The authors declare that the research was conducted in the absence of any commercial or financial relationships that could be construed as a potential conflict of interest.

## Supplementary Material

FIGURE S1 A timeline of the study. After adaptation for one week, 32 male rats were randomly divided into control group, CUMS group, SNI-sham group and SNI group. At day 0 and the end day, behavior tests were performed. And, weight gain and Von Frey test were measured per week.

FIGURE S2 The open field behavior in the total travel distance (A), the frequency of central crossing (B), number of rearing (C) and the duration of the central zone (D) before exposure to chronic stress. N = 8, no significance.
