# Supplementary figures and images for: Chronic Psychological Stress, but Not Chronic Pain Stress, Influences Sexual Motivation and Induces Testicular Autophagy in Male Rats
Source: Front Psychol. 2020 Apr 30;11:826. doi: 10.3389/fpsyg.2020.00826 (PMC7203493; doi:10.3389/fpsyg.2020.00826)

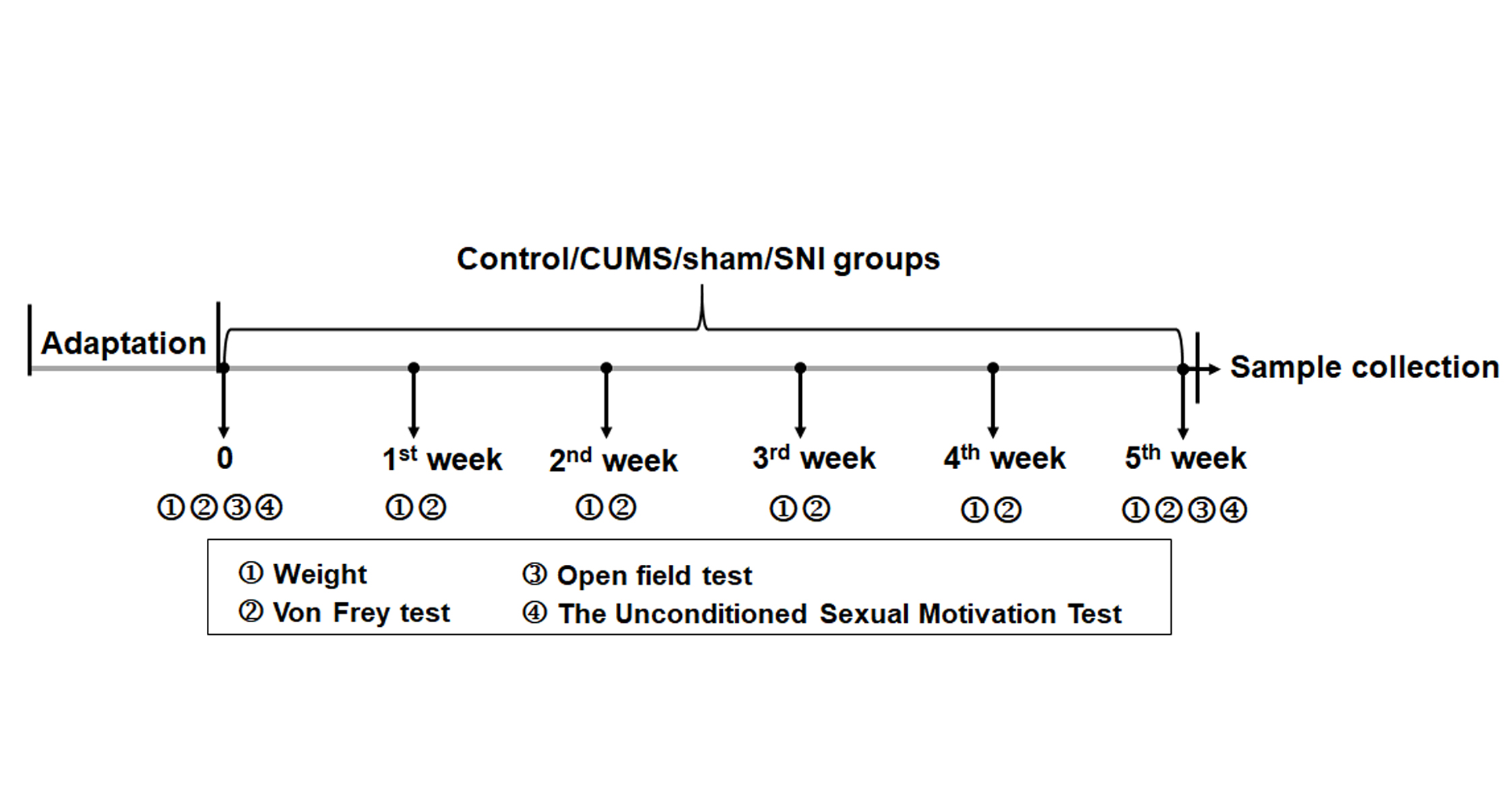

Supplement: FIGURE S1 — A timeline of the study. After adaptation for one week, 32 male rats were randomly divided into control group, CUMS group, SNI-sham group and SNI group. At day 0 and the end day, behavior tests were performed. And, weight gain and Von Frey test were measured per week. [file Image_1.JPEG]

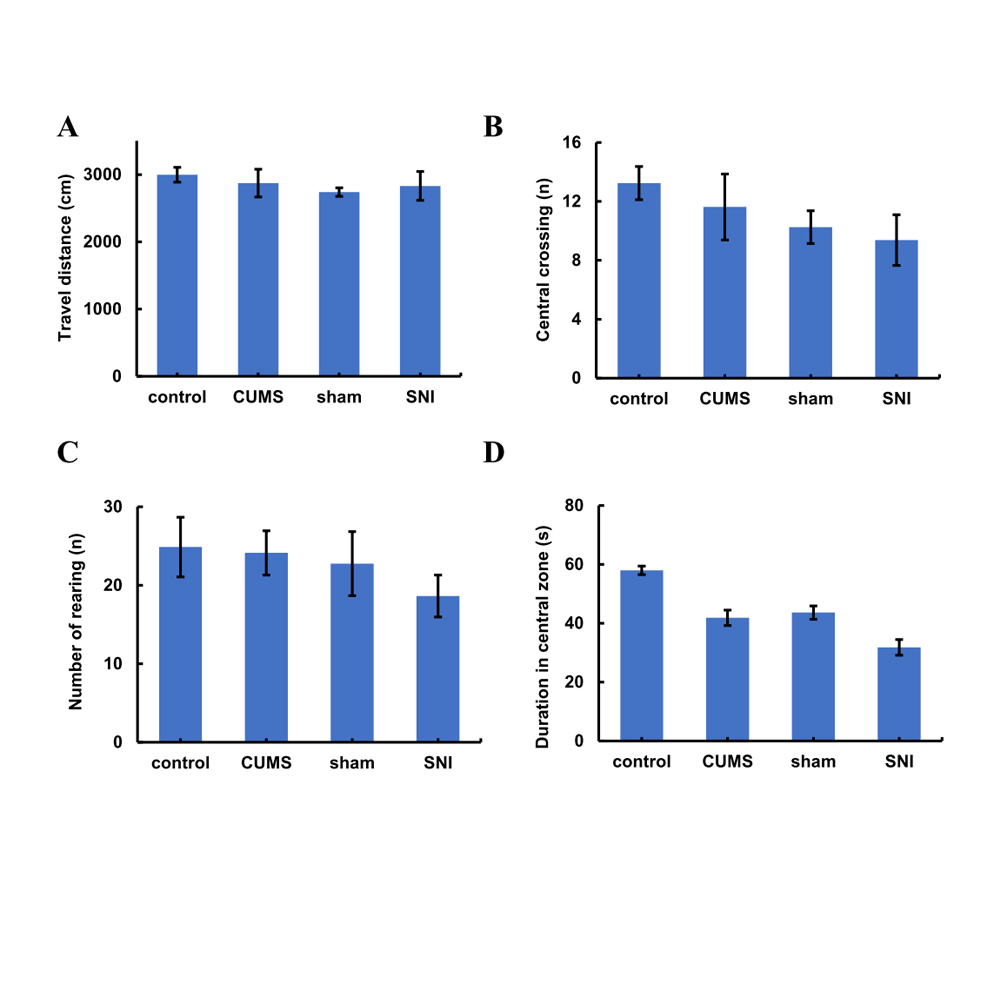

Supplement: FIGURE S2 — The open field behavior in the total travel distance (A), the frequency of central crossing (B), number of rearing (C) and the duration of the central zone (D) before exposure to chronic stress. N = 8, no significance. [file Image_2.JPEG]
